# Delayed presentation of a sigmoid colon injury following blunt abdominal trauma: a case report

**DOI:** 10.1186/1752-1947-6-247

**Published:** 2012-08-20

**Authors:** Gokhan Ertugrul, Murat Coskun, Mahsuni Sevinc, Fisun Ertugrul, Toygar Toydemir

**Affiliations:** 1 Department of General Surgery, Düzce Atatürk State Hospital, 81100, Muncurlu, Düzce, Turkey; 2 Department of Anaesthesiology and Reanimation, Düzce Atatürk State Hospital, Muncurlu, Düzce, Turkey; 3 Department of General Surgery, İstanbul Surgery Hospital, Nişantaşı, İstanbul, Turkey

**Keywords:** Abdominal trauma, Colon injury, Sigmoid loop colostomy

## Abstract

**Introduction:**

The low incidence of colon injury due to blunt abdominal trauma and the lack of a definitive diagnostic method for the same can lead to delays in diagnosis and treatment, subsequently resulting in high morbidity and mortality.

**Case presentation:**

A 66-year-old woman with sigmoid colon injury was admitted to our emergency department after sustaining blunt abdominal trauma. Her physical examination findings and laboratory results led to a decision to perform a laparotomy; exploration revealed a sigmoid colon injury that was treated by sigmoid loop colostomy.

**Conclusions:**

Surgical abdominal exploration revealed gross fecal contamination and a perforation site. Intra-abdominal irrigation and a sigmoid loop colostomy were performed. Our patient was discharged on post-operative day six without any problems. Closure of the sigmoid loop colostomy was performed three months after the initial surgery.

## Introduction

The low incidence of colon injury due to blunt abdominal trauma and the lack of a definitive diagnostic method for the same can lead to delays in diagnosis and treatment, subsequently resulting in high morbidity and mortality.

We present the case of a woman who presented with blunt abdominal trauma following a fall who was admitted to our emergency department; sigmoid loop colostomy was performed due to sigmoid colon injury. We compare the outcome in our patient with those reported in the literature.

## Case presentation

**Figure 1:**
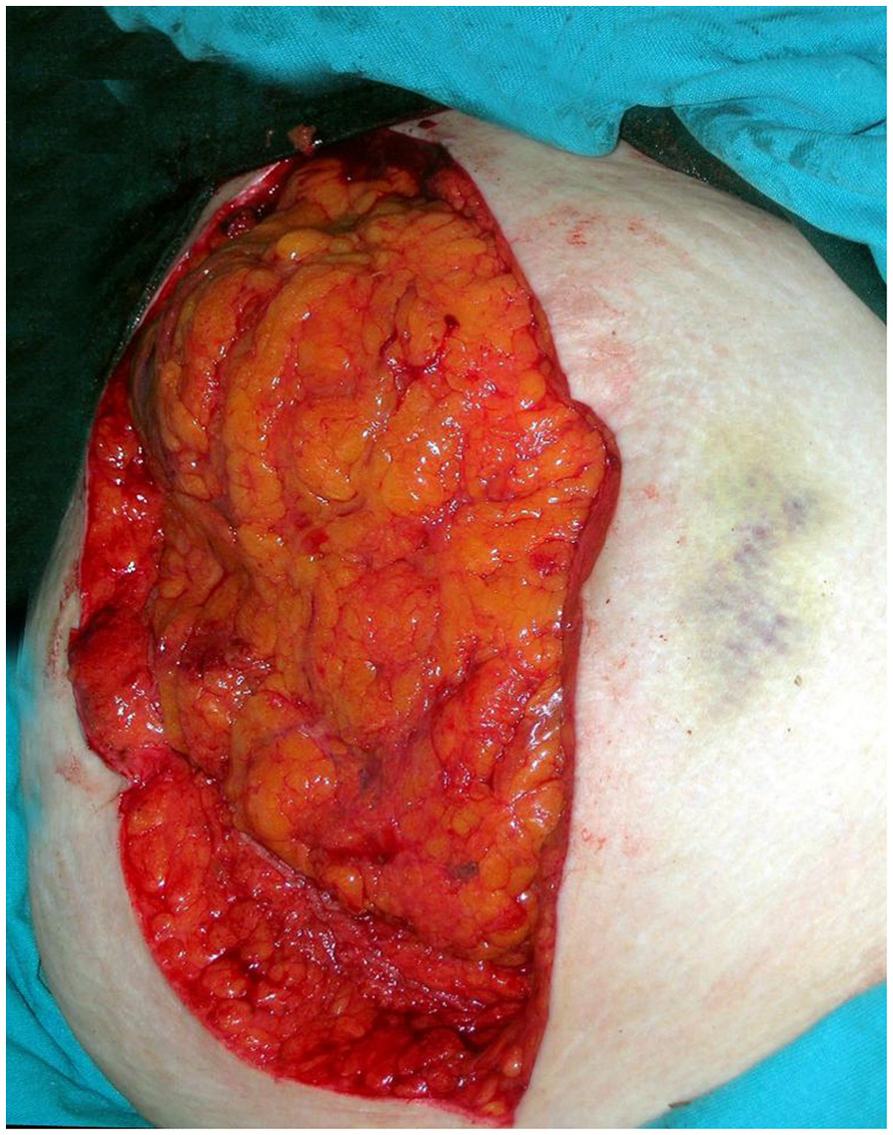
Ecchymosis around the umbilicus.

**Figure 2:**
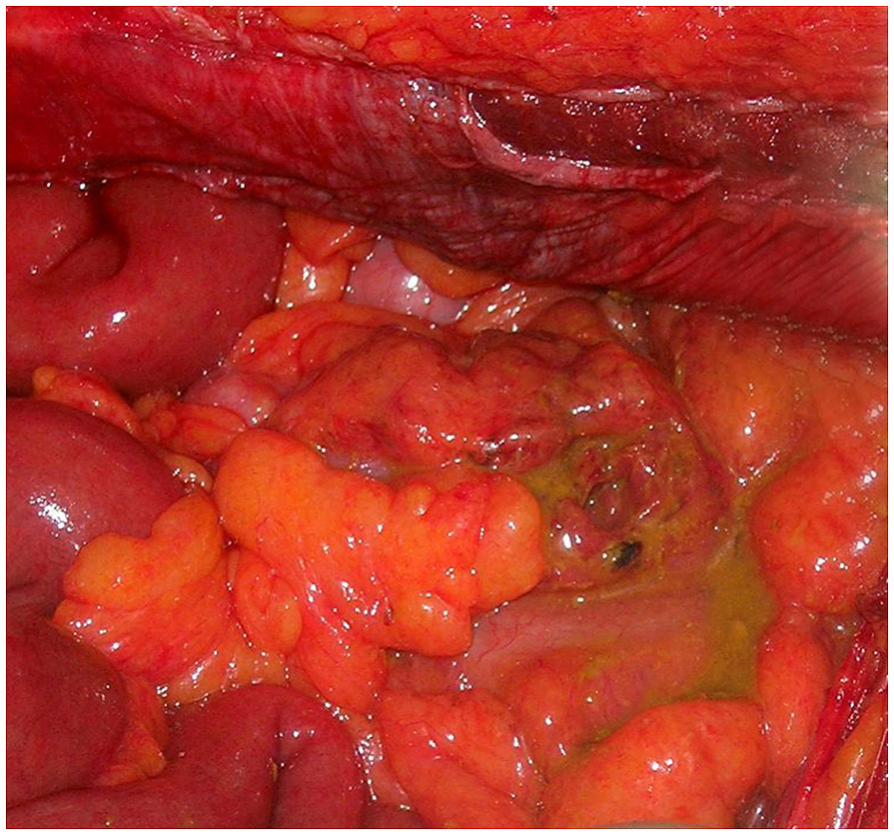
Fecal contamination.

**Figure 3:**
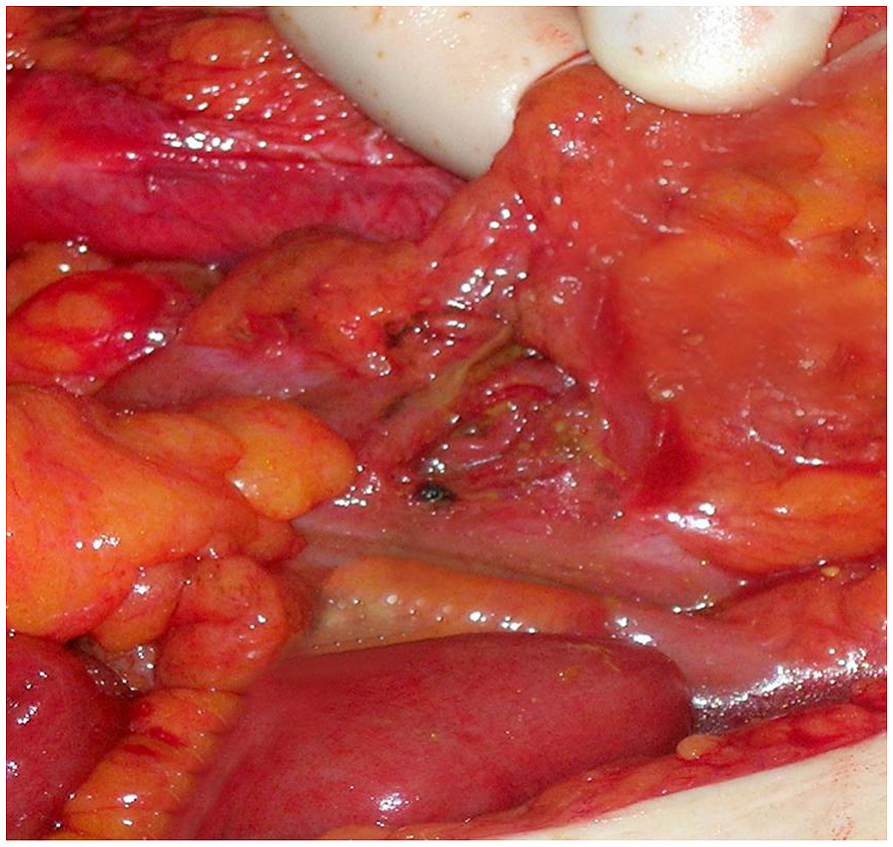
Sigmoid colon perforation.

A 66-year-old woman was admitted to our emergency department with complaints of abdominal pain, nausea and vomiting. Our patient had fallen at home 10 days ago. A physical examination revealed ecchymosis around the umbilicus (Figure 1), and tenderness, muscular rigidity, rebound tenderness, and minimal distension in all quadrants. Laboratory findings were normal apart from her leukocyte count (15.2 × 10^3^ cells/μL), hemoglobin (9.9g/dL) and hematocrit (29.3%) levels. An upright plain abdominal X-ray revealed multiple air-fluid levels. Our patient also had hypertension, and she was taking an angiotensin II receptor antagonist once daily. Surgical abdominal exploration revealed gross fecal contamination and a perforation site (Figure 2). The perforation site was in the anti-mesenteric side of the proximal sigmoid colon; it was approximately 2cm in size and had a coagulum around it (Figure 3). Intra-abdominal irrigation and a sigmoid loop colostomy were performed. Our patient was discharged on post-operative day six without any problems. Closure of the sigmoid loop colostomy was performed three months after the initial surgery. Colonoscopy that was performed post-operatively did not reveal diverticula in any of the segments of the colon. At post-operative month 12, our patient did not have any complaints.

## Discussion

Colon injuries generally occur after penetrating abdominal trauma, whereas they are rarely encountered after blunt abdominal trauma. In a retrospective study, the incidence of colon injuries due to blunt abdominal trauma has been reported to be 1.1% [1]. Motor vehicle accidents are the most common reason of colon injuries due to blunt abdominal trauma [2]. Other common causes include impacts to the abdomen (a direct blow, occupational accidents) and falls [3]. The retrospective study conducted by Carrillo *et al.* on 27 patients showed that 20 patients (74%) presented due to intra-vehicular accident, five patients (18.5%) presented due to extra-vehicular accident, one patient (3.7%) presented due to assault, and one patient (3.7%) presented due to boating accident [4]. In a retrospective study conducted by Zheng *et al.* on 82 patients, 57 patients (69.5%) presented due to an out-of-vehicle traffic accident, 18 patients (21.9%) presented due to an occupational accident, six patients (7.3%) presented due to an assault, and one patient (1.2%) presented due to an explosion [5]. The retrospective study conducted by Öztürk *et al.* on 64 patients revealed that 32 patients (50%) presented due to motor vehicle accident, 13 patients (20.3%) presented due to fall from height, 12 patients (18.7%) presented due to car crashing, and seven patients (10.9%) presented due to assault [6]. Barden *et al.* reported bull butting in one patient [7], whereas Ceci *et al.* reported intra-vehicular accident in one patient [8]. In the present case our patient had injured her abdominal region in the bathroom due to a fall.

Several mechanisms have been described for colon injuries occurring after blunt abdominal trauma. Crushing of the colonic segment between two objects (between the seat belt and vertebra or pelvis posteriorly) is the most widely accepted mechanism [9]. This results in local lacerations of the bowel wall, mural and mesenteric hematomas, transection of the bowel, localized devascularization and full-thickness contusions. Devitalization of the areas of contusion may subsequently result in late perforation. Rapid deceleration is the second mechanism. This creates shearing forces between the natural fixed points, which are the Treitz ligament, both ends of the sigmoid colon, and ileocecal junction, and the mobile portions of the colon. The third mechanism is a burst injury, which occurs by the closure of the colonic segments during trauma. The bowel ruptures or bursts when the intra-luminal pressure exceeds the tensile strength of the bowel wall [9]. The transverse colon is the most vulnerable colonic segment to blunt trauma due to its unprotected location [2]. The sigmoid colon is relatively less vulnerable and is generally exposed to closed-loop perforations [2]. Carrillo *et al.* reported sigmoid colon injury in 12 patients (44.4%), right colon and caecum injury in eight patients (29.6%), transverse colon injury in five patients (18.5%), and rectum injury in two patients (7.4%) [4]. In the study conducted by Zheng *et al.*, transverse colon injury was seen in 32 patients (39%), right colon injury was seen in 21 patients (25.6%), descending colon injury was seen in 16 patients (19.5%), sigmoid colon injury was seen in 13 patients (15.8%), and rectum injury was seen in five patients (6%) [5]. A total of 30 patients (46.8%) had left colon injury, 17 patients (26.6%) had right colon injury, and 17 patients (26.6%) had transverse colon injury in the study conducted by Öztürk *et al.*[6]. In their studies, Barden *et al.* reported transverse colon injury [7] and Ceci *et al.* reported sigmoid colon injury [8]. In the present case our patient had a sigmoid colon injury.

Isolated colon injury following blunt abdominal trauma is a rarely encountered condition. Colon injury is usually accompanied by other intra-abdominal organ injuries, with the small intestine, spleen, liver and pancreas being the leading areas. Isolated colon injury was detected in two patients, whereas colon injury was accompanied by liver injury in 16, spleen injury in 13, and small intestine injury in 12 patients, in the study conducted by Carrillo *et al.*[4]. Zheng *et al.* detected isolated colon injury in 20 patients and reported that accompanying injuries were in turn localized to the small intestine, spleen, liver, and kidney [5]. Isolated colon injury was detected in seven patients in the study conducted by Öztürk *et al.*, in which colon injuries were most commonly accompanied by small intestine, spleen, liver, and pancreas injuries [6]. Barden *et al.* detected transverse colon injury [7], whereas Ceci *et al.* detected sigmoid colon injury [8]. We found no accompanying further intra-abdominal organ injury in our patient’s case.

In a patient thought to have a colon injury caused by blunt abdominal trauma, the time between emergency department admission and surgery is of particular importance. A shorter duration minimizes the morbidity and mortality that would be encountered in the post-operative period. The rate of complications associated with colon injury is significantly higher if the duration is longer than 24 h after the injury [10].

At present, there is no single method to accurately diagnose colon injuries caused by blunt abdominal trauma. There are some studies suggesting the efficacy of repetitive physical examination and observation in diagnosing colon injury caused by blunt abdominal trauma in the first six hours, during which the signs of peritoneal irritation appear [11]. Tenderness, guarding, distension and abdominal wall contusion are valuable findings on physical examination. However, the absence of these findings does not rule out intra-abdominal pathology. The presence of leukocytosis becomes significant when interpreted together with the findings from physical examinations and the results of other diagnostic methods [12]. Plain radiographs are not reliable in detecting the presence of a significant injury; the results appear normal in most cases [13]. Ultrasonography has been widely used to evaluate blunt abdominal trauma [14]. Ultrasonographic findings of free fluid in the abdomen, particularly between the intestinal loops without the presence of solid organ injury, may indicate a bowel injury. Computed tomography is the most appropriate diagnostic tool to document abdominal injury; however, its diagnostic value for patients with colon injury remains controversial. On computed tomography, presence of free air in the abdomen and extravasation of the contrast agent are significant findings [15].

Treatment options include primary closure (colorrhaphy), resection with anastomosis, and colostomy. Primary closure (colorrhaphy) is performed for injuries involving less than 50% of the colonic wall, whereas resection with anastomosis is performed when the tissue loss is more than 50% or when there is extensive mesenteric injury impairing the blood supply [16]. Colostomy should be performed when there are more than two abdominal organ injuries, when the amount of intra-abdominal bleeding is above 1000mL, when there is gross fecal contamination within the abdomen, and when the time between the injury and treatment exceeds eight hours [17-20]. Carrillo *et al.* performed resection plus ostomy in 12 patients (44.4%), resection plus anastomosis in 11 patients (40.7%), and primary repair in four patients (14.8%) [4]. Zheng *et al.* performed primary repair in 67 patients (81.7%), and ostomy in 15 patients (18.2%) [5]. Öztürk *et al.* performed primary repair in 40 patients (62.5), resection plus anastomosis in 13 patients (20.3%), and ostomy in 11 patients (17.1%) [6]. Primary repair was performed in the study conducted by Barden *et al.*[7], whereas resection plus anastomosis were performed in the study conducted by Ceci *et al.*[8]. In the present study, we performed a sigmoid loop colostomy in our patient. Primary closure and resection with anastomosis have been the choice of treatment within the last two decades as they are associated with reduced morbidity, mortality and cost.

Morbidity and mortality rates following blunt abdominal traumas are increased in colon injuries depending on the difficulties in diagnosis and treatment. Morbidity was observed in 12 (44.4%) and mortality was observed in 10 (37%) patients in the study conducted by Carrillo *et al.*[4]. Zheng *et al.* reported morbidity in 17 (20.7%) and mortality in five (6.1%) patients [5]. Again, Öztürk *et al.* reported morbidity in 17 (26.5%) patients, whereas mortality was observed in seven (10.9%) patients [6]. The most common post-operative complications were wound site infection, intra-abdominal abscess, intra-abdominal sepsis, and post-operative bleeding. No morbidity or mortality was observed after the surgery in our patient’s case.

## Conclusions

In summary, colon injury due to blunt abdominal trauma is a rare clinical condition, and treatment delays due to difficulties in diagnosis increase the morbidity and mortality rates.

## Consent

Written informed consent was obtained from the patient for publication of this case report and any accompanying images. A copy of the written consent is available for review by the Editor-in-Chief of this journal.

## Competing interests

The authors declare that they have no competing interests.

## Authors’ contributions

GE, MC and FE operated on our patient. GE, MS and TT analyzed and interpreted the data from our patient regarding the blunt abdominal trauma and colonic injury. GE was a major contributor in writing the manuscript. All authors read and approved the final manuscript.
